# Thin-film synthesis of superconductor-on-insulator A15 vanadium silicide

**DOI:** 10.1038/s41598-021-82046-1

**Published:** 2021-01-27

**Authors:** Wenrui Zhang, Anthony T. Bollinger, Ruoshui Li, Kim Kisslinger, Xiao Tong, Mingzhao Liu, Charles T. Black

**Affiliations:** 1 Center for Functional Nanomaterials, Brookhaven National Laboratory, Upton, NY 11973 USA; 2 Condensed Matter Physics and Materials Science Division, Brookhaven National Laboratory, Upton, NY 11973 USA; 3 Department of Chemistry, Stony Brook University, Stony Brook, NY 11794 USA

**Keywords:** Superconducting properties and materials, Quantum information, Electronic devices

## Abstract

We present a new method for thin-film synthesis of the superconducting A15 phase of vanadium silicide with critical temperature higher than 13 K. Interdiffusion between a metallic vanadium film and the underlying silicon device layer in a silicon-on-insulator substrate, at temperatures between 650 and 750 °C, favors formation of the vanadium-rich A15 phase by limiting the supply of available silicon for the reaction. Energy dispersive X-ray spectroscopy, X-ray photoelectron spectroscopy, and X-ray diffraction verify the stoichiometry and structure of the synthesized thin films. We measure superconducting critical currents of more than 10^6^ amperes per square centimeter at low temperature in micron-scale bars fabricated from the material, and an upper critical magnetic field of 20 T, from which we deduce a superconducting coherence length of 4 nm, consistent with previously reported bulk values. The relatively high critical temperature of A15 vanadium silicide is an appealing property for use in silicon-compatible quantum devices and circuits.

## Introduction

In the early 1950s, the cubic A15 phase (A_3_B) was discovered to be superconducting, with the notable example of A15 vanadium silicide (V_3_Si) briefly having the highest known critical temperature (*T*_*c*_) of any material (17.1 K)^1,2^. Other A15 superconductors were soon found to have higher *T*_*c*_ [e.g., Nb_3_Sn (18 K)^3^, Nb_3_Ge (23 K)^4^], and the later discovery of cuprate perovskites^5,6^ heralded an era of intense study of so-called high-*T*_*c*_ superconductivity—leaving the material V_3_Si largely unexplored. As a silicide, V_3_Si is also appealing for its compatibility with silicon technology. In microelectronic devices, transition metal silicides are thoroughly studied and used for low-resistance metallic contacts to field-effect transistors^7–10^. V_3_Si is an *s*-wave, type-II superconductor with isotropic energy gap, suitable for superconducting devices such as Josephson junctions and superconducting quantum interference devices (SQUIDs)^11,12^. Furthermore, geometrical lattice matching analysis shows that V_3_Si forms a high-quality, epitaxial interface with Si with very small lattice mismatch (0.4%) and common cell area (40 Å^2^)^13^.

In this work, we report a new method for synthesis of phase pure, superconducting V_3_Si thin films on silicon dioxide/silicon substrates (SiO_2_/Si)—a process amenable to fabrication of superconductor-on-insulator devices leveraging silicon technology, and potentially compatible with silicon CMOS fabrication. In the bulk, V_3_Si is typically synthesized by melting together a stoichiometric mixture (3:1) of elemental vanadium (V) and Si in an inert atmosphere^14^. Thin films have been previously prepared by vapor phase deposition, including electron-beam co-evaporation of V and Si^15^, magnetron sputtering from a stoichiometric target^16^, and molecular beam epitaxy^17^. In silicon microelectronics, transition metal silicides are formed by silicidation—the process of annealing metal films deposited on silicon^8^. However, silicidation of V deposited on Si typically forms the Si-rich vanadium silicide phase (VSi_2_), which is not superconducting, due to the excess supply of available Si during the reaction^18,19^. Silicidation of V deposited on thermally-grown SiO_2_ can form the A15 phase V_3_Si under the right conditions^18,19^, presumably because diffusion of Si out of SiO_2_ is slow enough to permit formation of the V-rich phase. However, this process also results in oxygen from the SiO_2_ reacting with V to form vanadium oxides that are mixed with V_3_Si in the final structure.

## Results and discussion

### Thin film synthesis

**Figure 1:**
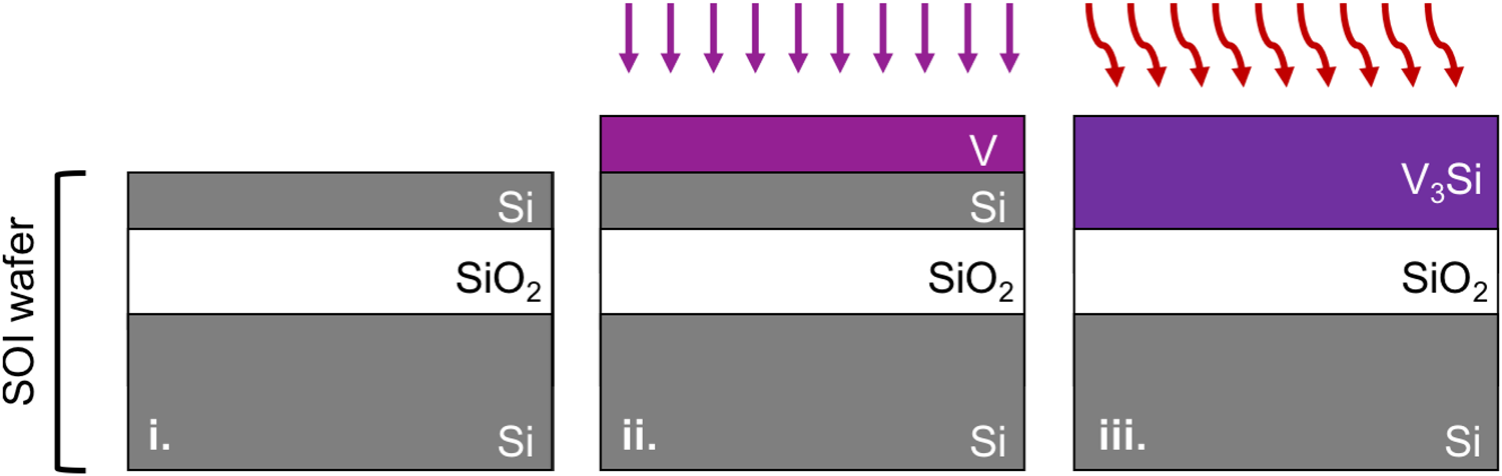
Thin-film synthesis of A15 vanadium silicide on insulator. (**i**) Silicon on insulator (SOI) substrate. (**ii**) Vanadium deposition by thermal evaporation. (**iii**) Silicidation by annealing in vacuum.

**Figure 2:**
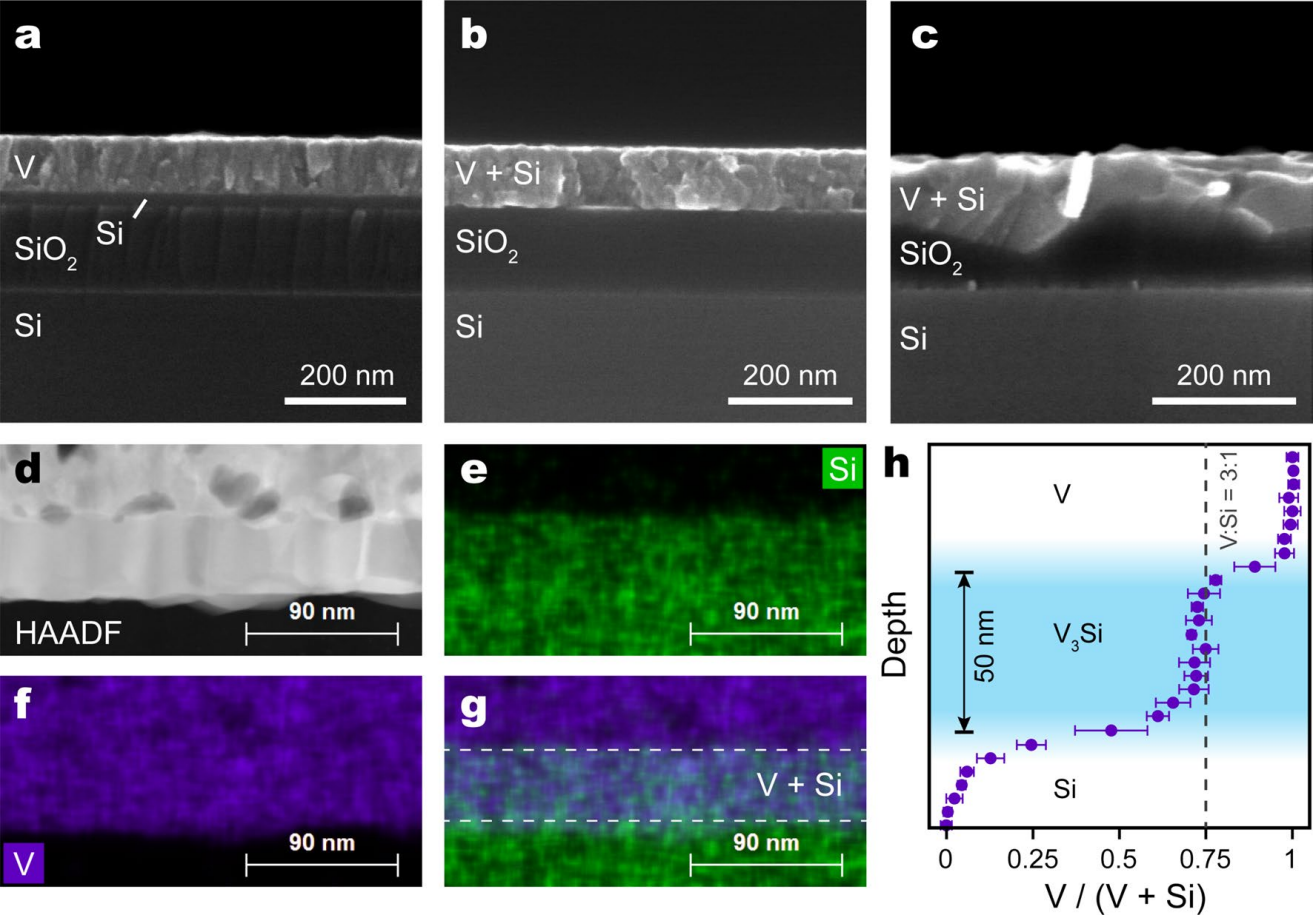
Cross-sectional SEM images of: (**a**) V film deposited onto a SOI substrate, and similar samples after annealing in vacuum at (**b**) 700 °C and (**c**) 900 °C. (**d**) HAADF STEM image of the film annealed at 700 °C. (**e**, **f**) EDX mapping of the area shown in (**d**), denoting location of (**e**) Si and (**f**) V. (**g**) Overlapped maps (**e**) and (**f**) show the vanadium silicide region contains both V and Si. (**h**) Quantitative EDX analysis shows a 3:1 V:Si ratio within the V_3_Si layer.

Here, we synthesize phase-pure, vanadium-rich V_3_Si thin films by silicidation, by reacting only the thin Si device layer of a silicon-on-insulator (SOI) wafer (Fig. 1). By optimizing the silicidation anneal conditions, we completely convert the Si device layer into V_3_Si without reacting with the underlying SiO_2_, leaving a sharp and smooth V_3_Si/SiO_2_ interface. In our experiments, we start with SOI wafers having a 20-nm Si (100)-oriented device layer on top of a 140 nm thick thermal SiO_2_. Stoichiometric conversion of the entire 20 nm thick Si layer to V_3_Si by silicidation requires the number of V atoms supplied by a 42 nm thick film (see “Methods” section). Most typically, we deposit 90 nm of V—which provides an oversupply of V to ensure complete silicidation of the Si device layer. Prior to annealing, we observe a sharp V/Si interface from cross-sectional scanning electron microscope (SEM) images (Fig. 2a). The samples are annealed in high vacuum (10^−7^ Torr) for 15 min, at temperatures (*T*) ranging from 650 °C to 900 °C. The V/Si interface disappears after annealing at sufficiently high temperature (Fig. 2b), due to V-Si interdiffusion. Silicidation at *T* = 800 °C or lower maintains a sharp, smooth interface at the underlying SiO_2_ layer, while higher *T* roughens this interface, indicating a reaction with the SiO_2_ (Fig. 2c).

**Figure 3:**
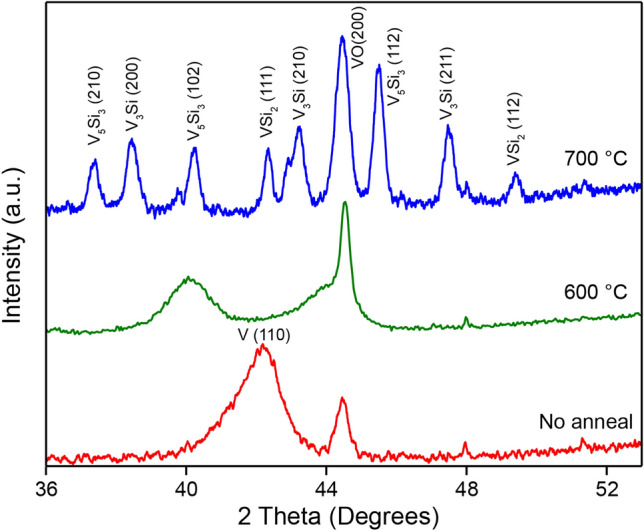
X-ray diffraction spectra (XRD) of V films deposited on SOI, before annealing, and after vacuum annealing at 600 °C and 700 °C. The pattern after annealing at 700 °C shows the formation of the A15 V_3_Si phase. The 600 °C pattern shows a transition from metallic V to vanadium silicide. Due to exposure to air during sample handling, vanadium oxide (VO) is also formed and evident in the diffraction patterns.

Scanning transmission electron microscopy (STEM) reveals more details of the silicidation process. High-angle annular dark field (HAADF) cross-sectional images of a sample annealed at 700 °C (Fig. 2d–f) show two distinct regions within the V-Si layer: an upper, porous region and an underlying, denser layer (Fig. 2d). Energy dispersive X-ray spectroscopy (EDX) shows that the top layer is pure V and the bottom layer contains both V and Si (Fig. 2e–g), consistent with our supplying excess V for the silicidation reaction. The porosity of the V layer is a result of deposition by thermal evaporation, as deposited V atoms quickly lose kinetic energy on the cold substrate and have insufficient mobility to form close-packed films. Quantitative analysis of the EDX maps shows that the bottom layer contains V and Si in a 3:1 ratio (Fig. 2h). X-ray diffraction (XRD) confirms formation of the A15 phase of V_3_Si for samples annealed at 700 °C, along with impurities of VSi_2_ and V_5_Si_3_ (Fig. 3). Annealing at 600 °C shifts the broad V (110) peak at 42° to 40°, which is matched to V_5_Si_3_ (102) but is very broad and likely reflects an intermediate amorphous phase formed during interdiffusion. The presence of VO (200) peak in all samples suggests partial oxidation of the deposited V due to air exposure. For any sample annealed at less than 800 °C, we do not expect reaction between vanadium and SiO_2_, which is evident from the sharp interface between the V/Si layer and the SiO_2_ layer (Fig. 2b). According to the EDX maps, the V_3_Si layer is about 50 nm in thickness, which matches closely the thickness expected from stoichiometric conversion of Si (52 nm, see “Methods” section). The dense V_3_Si layer suggests that V is the predominant diffusion species, i.e., that silicidation occurs primarily by V diffusing into the Si lattice. Significant Si diffusion would result in the silicide inheriting the porous appearance of the deposited V layer. Our observation is consistent with those of previous studies, which noted an ‘ordered Cu_3_Au rule’^20^, suggesting that majority atoms are the most mobile ones in the class of A_3_B compounds^21^.

**Figure 4:**
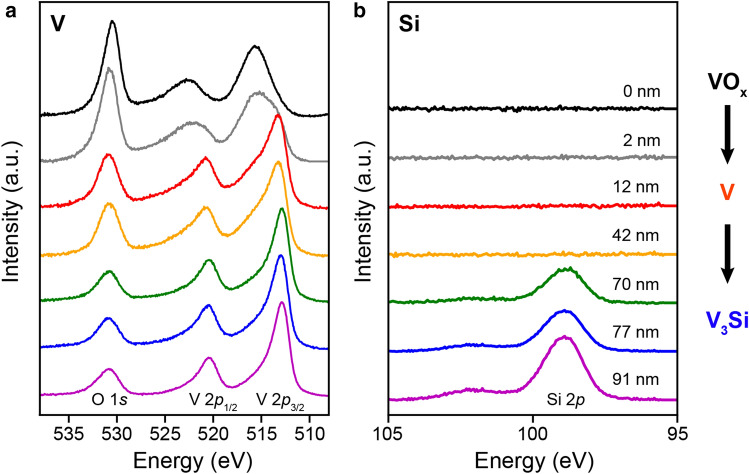
Depth-profiled X-ray photoelectron spectra of a V_3_Si thin film, probed from the film top surface (upper curve) toward the bottom (lower curve). (**a**) the V 2*p* and O 1*s* levels, and (**b**) the Si 2*p* level. Depth-profiling is carried out by Ar ion milling. The depth values are calculated by assuming a uniform ion mill rate. Offsets are applied for clarity of viewing.

Profiling the vanadium silicide film by sequential argon ion milling and X-ray photoelectron spectroscopy (XPS) verifies its composition (Fig. 4). The film top surface is composed of excess V that has been oxidized upon air exposure, with the V 2*p*_3/2_ peak positioned at 515.6 eV, corresponding to a V^3+^ oxidative state (Fig. 3a, black color). Beginning with spectra taken from 12 nm below the top surface, this peak shifts to 513 eV, which corresponds to elemental V (Fig. 4a, red color). The emergence of the Si 2*p* peak at 99.0 eV (Fig. 4b, green color) at a depth of 70 nm and beyond indicates the conversion to V_3_Si. In this region, the Si 2*p* peak position is very close to elemental Si (99.4 eV), while the V 2*p*_3/2_ peak remains at 513 eV, consistent with the metallic nature of V in A15 V_3_Si^22^. By analyzing the peak areas and sensitivity factors associated with V 2*p*_3/2_ and Si 2*p* elements in the V_3_Si region, at a depth of 77 nm (Fig. 4, blue color), the relative elemental ratio between V and Si is obtained at 3:1, consistent with EDX measurements (Fig. 2h). The O 1*s* peak at 531 eV across all depths indicates the presence of vanadium oxide (VO) in both metallic V and vanadium silicide region due to exposure to air.

### Transport measurements

**Figure 5:**
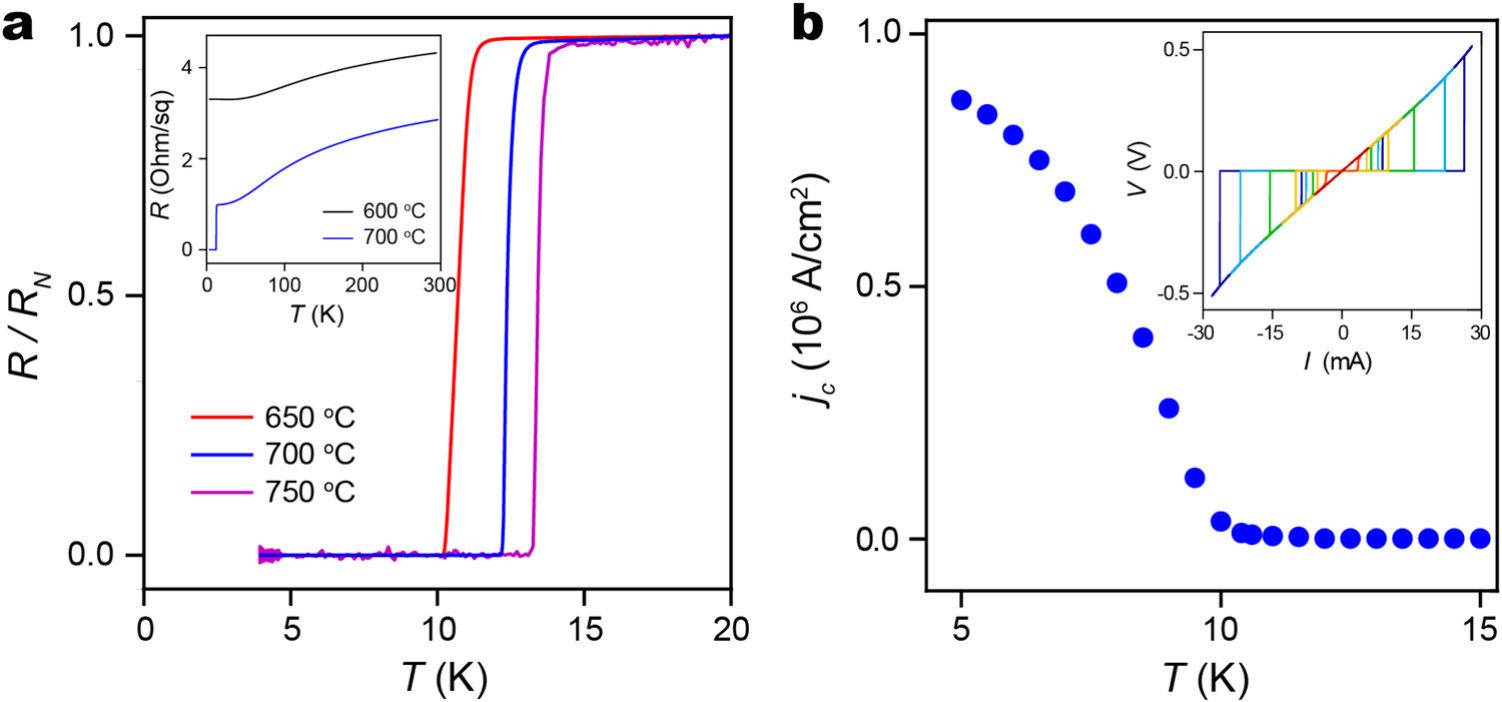
(**a**) Electrical resistance versus temperature with no magnetic field, for vanadium silicide films annealed at 650 °C (red), 700 °C (blue), and 750 °C (magenta), normalized against normal state resistance at 20 K (inset). Electrical resistance from room temperature to 4 K, for vanadium silicide films annealed at 600 °C (black) and 700 °C (blue). No superconductivity is observed in the 600 °C sample, consistent with the absence of a V_3_Si phase. (**b**) Critical current density (*j*_*c*_) of a 30 μm wide bar fabricated from V_3_Si thin film annealed at 700 °C (inset). Representative voltage-current curves used to determine the critical current density (*T* = 5–15 K).

Four-probe resistance measurements of V_3_Si films versus *T* reveal their metallic nature and the superconducting transition temperature (*T*_*c*_). We measure the film in-plane resistance using a constant-current bias of 50 µA and vary *T* at a rate of ~ 1 K min^−1^ with no magnetic field. The sheet resistance of a 50 nm thick V_3_Si film silicided at 700 °C decreases from 2.8 to 1.0 Ω/sq upon cooling from room *T* to 20 K, a resistance ratio of 2.8 (Fig. 5a, inset). These values reflect the parallel normal state resistance of both the V_3_Si and the overlaying metallic V layer (also about 50 nm thick). At *T*_*c*_ = 13.75 K, the film resistance abruptly decreases to zero with a transition width of 0.3 K, marking the onset of superconductivity. Films silicided at *T* between 650 and 750 °C all showed superconducting transitions, with *T*_*c*_ decreasing with decreasing silicidation *T* (Fig. 5a). We do not observe a superconducting phase transition in films silicided at *T* ≤ 600 °C measured to temperatures as low as 4 K (Fig. 5a, inset). The critical current density is determined from voltage-current measurements of strips fabricated from V_3_Si thin film annealed at 700 °C. The strips are 30 µm in width and are patterned using photolithography, with etching performed in an argon ion mill (2.5 mA/cm^2^ beam density), and electrical contacts deposited by e-beam evaporation of 10 nm titanium and 300 nm gold. The voltage-current behavior of strips patterned from a V_3_Si film change from linear behavior above *T*_c_ to non-linear near the superconducting transition (Fig. 5b, inset). Films display sharp, fully developed, critical currents at *T* < *T*_c,_ which increase as *T* is further reduced. At *T* = 5 K, the critical current density (*j*_*c*_) is 8.7 × 10^5^ A cm^−2^, slightly higher than previous reports taken on V_3_Si single crystals^23^. This is likely due to the presence of impurity phases acting as flux-pinning centers, which lead to the observed higher critical current densities. Because the *T*_*c*_ of metallic V is 5.4 K for single crystals^24^, and lower for amorphous films deposited from the vapor phase^25^, the only contributions from the 50-nm-thick amorphous V film to *j*_*c*_ that we would expect within the range of measured temperatures (5–15 K) is a proximity effect induced superconducting layer near the V_3_Si that serves to increase the effective thickness that carries supercurrent, which is taken into account in the main panel of Fig. 5b.

**Figure 6:**
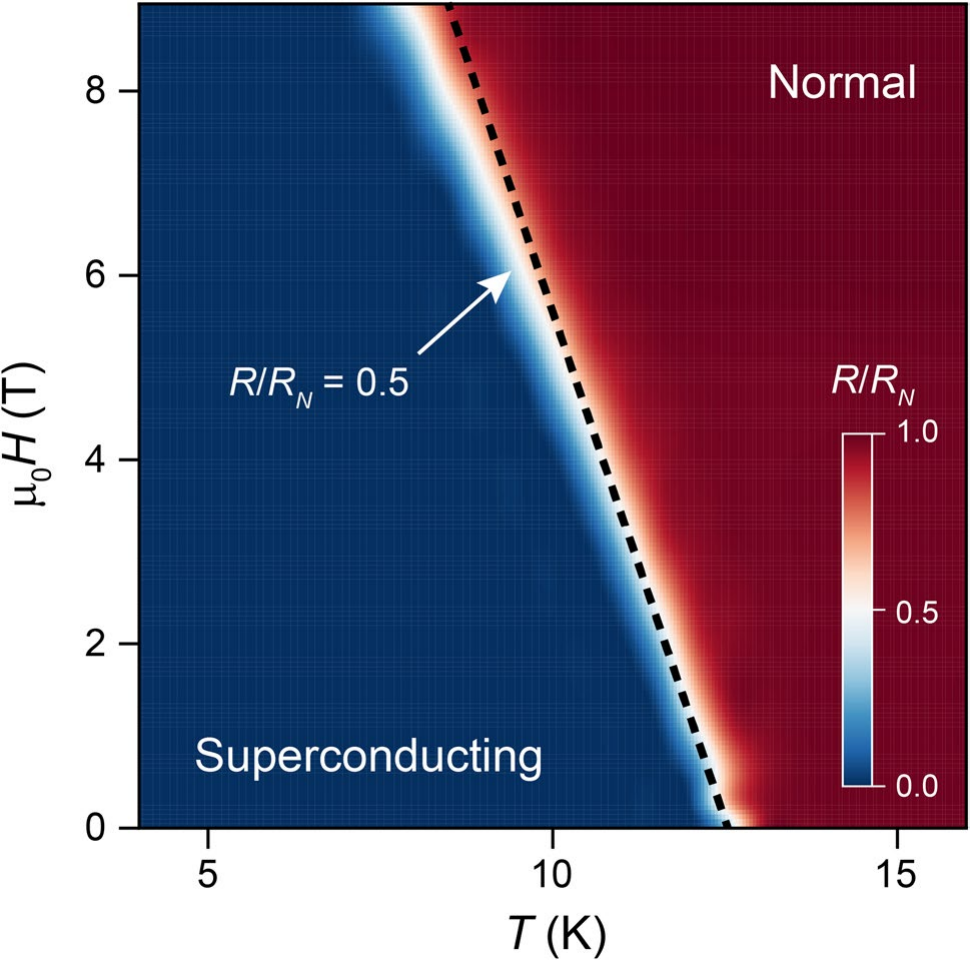
Magnetic field dependence of superconducting critical temperature. The white region separating the normal state (red) and superconducting state (blue) are separated by a thin white region denoting the upper critical field, *H*_c2_(*T*). A linear fit to *H*_c2_(*T*) near zero applied field (dashed line) is used to calculate *H*_c2_(0) using Eq. (1).

The A15 phase of vanadium silicide is a known type II superconductor that undergoes phase transitions at two different applied magnetic fields. At a lower critical magnetic field (*H*_*c*1_) the superconducting order parameter is locally suppressed and the material supports magnetic vortices, and at an upper critical field (*H*_*c*2_) the material reverts to the normal state. We measured the V_3_Si film resistance versus *T* in a series of increasing applied magnetic fields (*H*) in order to determine *H*_*c*2_(0), the upper critical field at 0 K. The *T*_*c*_ of a V_3_Si film silicided at 700 °C and having *T*_*c*_(0) ~ 13 K is continuously suppressed with increasing magnetic field (Fig. 6). For each *H*, we determine the corresponding *T*_*c*_(*H*) using the criteria *R*/*R*_*N*_ = 0.5 (midpoint *T*_*c*_), where *R*_*N*_ is the normal state resistance. In the color map shown in Fig. 5, the dependence of *H*_*c*2_ on *T* appears as a narrow white region separating normal and superconducting behaviors. Because the highest magnetic fields available in our experiment (9 T) are insufficient to completely suppress superconductivity in our films, we deduce *H*_*c*2_(0) for the material indirectly using the Werthamer-Helfand-Hohenberg model, which gives:

1 $$H_{c2} \left( 0 \right) = - \frac{{\pi^{2} T_{c} \left( 0 \right){ }}}{{8e^{\gamma } }}\left. {\frac{{dH_{c2} }}{dT}} \right|_{{T = T_{c} \left( 0 \right){ }}} ,$$

where $\gamma \approx 0.5772$ is Euler’s constant^26^. A linear fit to the *H*_*c*2_ – *T* relation in the range of 0 T < *μ*_0_*H*_*c*2_ ≤ 1 T provides both the tangent term in Eq. (1) and zero field *T*_*c*_(0), respectively, as the slope and the *T*-axis intercept (Fig. 5). From this analysis, we obtain *μ*_0_*H*_*c*2_(0) = 20 T, which is consistent with the previously-reported bulk value of *H*_*c2*_ = 23.5 T for V_3_Si^27^. Knowing ${H}_{c2}\left(0\right)$, we can also calculate the superconducting coherence length $\xi \left(0\right)$ in our V_3_Si to be 4 nm, using the relation ${{\mu }_{0}H}_{c2}\left(0\right)= {\Phi }_{0}/2\pi {\xi }^{2}\left(0\right)$^28^, where ${\Phi }_{0}=h/2e$ is the magnetic flux quantum, also consistent with previous reports^29^.

## Summary and outlook

In summary, we have described a new method for synthesis of the A15 phase of vanadium silicide by silicidation of metallic vanadium layers deposited on silicon-on-insulator substrates. The limited supply of silicon available for silicidation facilitates formation of the superconducting, vanadium-rich A15 phase. V_3_Si thin films synthesized on silicon-on-insulator substrates are type-II superconductors with *T*_*c*_ > 13 K. The relatively high *T*_*c*_ is an appealing property for use in silicon-compatible quantum devices and circuits.

## Methods

### Fabrication of V_3_Si thin films

The substrates, which are silicon-on-insulator (SOI) wafers with a 20 nm Si device layer and a 140 nm oxide SOI wafer, are dipped in a 10:1 buffered oxide etch (BOE) for 1 min to remove the native silicon oxide. Immediately after the oxide stripping, vanadium layers of different thicknesses are deposited by thermal evaporation in high vacuum (~ 10^−7^ Torr). After metal deposition, the substrate is annealed in high vacuum for 15 min, at temperatures ranging from 650 °C to 900 °C, to form V_3_Si by V-Si interdiffusion.

### Vanadium thickness for 3:1 V:Si stoichiometry

The molar volume (*V*_*m*_) of a substance is given by ${V}_{m}=M/\rho$, where *M* and *ρ* are, respectively, the substance’s molar mass and volumetric mass density. For crystalline Si, *M*_Si_ = 28.085 g mol^−1^ and *ρ*_Si_ = 2.33 g cm^−3^, which gives *V*_*m*, Si_ = 12.05 cm^3^ mol^−1^. For V metal, *M*_V_ = 50.94 g mol^−1^ and *ρ*_V_ = 6.0 g cm^−3^, which gives *V*_*m*, V_ = 8.49 cm^3^ mol^−1^. As such, the stoichiometric conversion of one unit volume of Si to V_3_Si requires 3 × *V*_*m*, V_/*V*_*m*, Si_ = 2.11. For a 20 nm layer of Si, this corresponds to 42 nm of V. After the conversion, with ${M}_{{\mathrm{V}}_{3}\mathrm{Si}}=180.9$ g mol^−1^ and ${\rho }_{{\mathrm{V}}_{3}\mathrm{Si}}=5.77$ g cm^−3^, a similar calculation gives an expected thickness of 52 nm for the V_3_Si layer.

### Scanning transmission electron microscopy (STEM) imaging

The specimen for STEM studies is a 40 nm-thick vertical cross-sectional slice of the silicide film mounted on a copper grid using the in situ focused ion beam milling and lift-out technique (Helios 600 dual beam FIB). High-angle annular dark field (HAADF) and energy dispersive X-ray spectroscopy (EDX) mapping images are collected over the same area simultaneously, using an FEI Talos F200X microscope.

### Transport measurements on V_3_Si thin films

Electrical transport measurements are performed in either a closed-cycle cryocooler or a top loading He-3 cryostat equipped with a 9 T magnet. Resistance is determined by delta mode averaging with a DC bias current provided by a source meter and sample voltage measured with a nanovoltmeter. Measurements of the *T*-dependence of the upper critical field are made by constant field temperature sweeps and verified by constant temperature magnetic field sweeps. The critical current density is determined from voltage-current measurements of strips fabricated from V_3_Si thin film annealed at 700 °C. The strips are 30 µm in width and are patterned by standard photolithography, with etching performed in an argon ion mill (2.5 mA/cm^2^ beam density), and electrical contacts deposited by e-beam evaporation of 10 nm Ti and 300 nm Au, followed by liftoff.
